# Preliminary Comparative Anatomical and Compositional Screening of the Pressed Fiber Fraction of Soybean (*Glycine max* L.) Green Biomass from Different Varieties Under a Single-Year Experimental Condition

**DOI:** 10.3390/plants15182750

**Published:** 2026-09-08

**Authors:** Szilvia Kovács, Rebeka Aszalósné Balogh, Bence József Eged, Attila Péter Kiss, Miklós Gábor Fári, Gábor Csatári

**Affiliations:** 1 Institute of Applied Plant Biology, Faculty of Agricultural and Food Sciences and Environmental Management, University of Debrecen, Böszörményi Street 138, 4032 Debrecen, Hungary; szkovacs@agr.unideb.hu (S.K.); egedbence@gmail.com (B.J.E.); fari@agr.unideb.hu (M.G.F.); csatari.gabor@agr.unideb.hu (G.C.); 2 Department of Food Biotechnology, Albert Kázmér Faculty of Agricultural and Food Sciences, Széchenyi István University, 9026 Gyor, Hungary; kiss.attila.peter@sze.hu

**Keywords:** soybean, pressed fiber, glucan, xylan, arabinan, Klason lignin, photosynthetic pigment, mineral elements

## Abstract

The present study aimed to characterize soybean pressed fiber obtained through wet fractionation of green biomass and evaluate the effects of variety and vegetation window on its composition and valorization potential. Four soybean varieties (Advisor, Bólyi 612, Isidor, and Pannónia kincse) were investigated across two distinct vegetation windows using anatomical, cell wall, biochemical, and pigment analyses. Significant variety-dependent differences were observed in several anatomical traits related to biomass quality. Isidor exhibited the greatest leaf blade thickness, while Advisor showed favorable stem structural characteristics and enhanced cuticle development. Cell wall analysis revealed significant differences in xylan, arabinan, and Klason lignin between vegetation windows, while glucan content was affected by both variety and vegetation window, with the highest value observed in Advisor during the first vegetation window. Photosynthetic pigment composition remained relatively stable among varieties and vegetation windows. Because the two vegetation windows occurred during different calendar periods, the observed differences may reflect both plant developmental status and seasonal environmental conditions and cannot be exclusively attributed to harvest timing. This one-year exploratory study provides baseline information on soybean pressed fiber as a potential secondary biomass resource. Further multi-year and multi-environment studies are required to assess the stability and environmental consistency of these findings.

## 1. Introduction

As the population of Earth exhibits dynamic growth, today’s agricultural practices face various challenges. Rapidly expanding food demands lead to an increased role of the about 990 million tonnes of agricultural waste and by-products generated each year [1,2]. By-products can be processed by means of circular economy systems based on sustainability needs. In circular economics, the basic aim is to recycle waste products back into the production cycle. By using the materials of the by-product or end-of-life product, a value-added product is gained, while cost-efficiency is improved and environmental impact is reduced [3,4,5]. The application of green biorefinery systems can be integrated into a circular agroeconomy [6].

As defined by the International Energy Agency, biorefining is a sustainable, broad-spectrum processing method, which turns biomass into value-added products such as food and feed components, biofuels, and chemicals [7]. According to Van ree et al. [8], three sub-systems can be distinguished within biorefinery systems: ‘Whole crop biorefineries’ (WCBs), which process only agricultural feedstocks; ‘Lignocellulosic feedstocks biorefineries’ (LCFBs), which process lignocellulosic residues; and ‘green biorefineries’ (GBs), which process immature cereals and herbaceous crops.

The previously mentioned wet fractionation is one of the biotechnological processes of lignocellulosic green biomass [9,10,11], which can yield valuable pressed fibers as a by-product (about 25–32% of the total mass). The main constituents of the obtained fibers are lignin, cellulose and hemicellulose [12,13,14,15]. The building blocks of these constituents can provide many industrial sectors with raw materials [9]. For example, the production of several natural sweeteners, as well as functional foods, which are becoming increasingly popular today, is based on such biotechnological processes [16,17]. The gained fiber constituents can also serve as excellent raw materials for the energy industry, producing bioethanol and biogas, which could result in a reduced use of fossil fuels [18,19,20,21]. The referred components are also used in the pharmaceutical industry and in the paper industry [22,23]. In addition, their use as animal feed is, of course, widespread [24]. In green biorefineries, the fiber-rich press cake generated after wet fractionation should be regarded as a valuable secondary raw material rather than a waste stream. Previous studies demonstrated that this fraction can be valorized through several routes, including ensiling for ruminant feed, microbial fermentation, and anaerobic digestion, as thermal-insulation materials, and for the recovery of cellulose, lignin and chlorophyll pigments for high-value biobased products. Integrated processing concepts have also shown that the residual press fractions retain most of the original organic matter, allowing efficient methane production or further material utilization. Ensiling has additionally been proposed as an effective strategy for ensuring year-round feedstock availability, although it may alter protein composition and therefore should be considered as part of the overall processing chain [25]. Fibers may also retain biologically relevant minerals and other bioactive compounds. The nutritional availability of mineral elements from dietary fiber can be influenced by processing conditions and the presence of antinutritional factors such as phytic acid and tannins [26,27,28,29,30]. Therefore, the mineral composition of plant-derived fiber fractions may contribute to their potential value as food or feed ingredients and warrants consideration in the context of biomass valorization [31].

Soybeans are grown worldwide mainly for their seeds, whose quality is determined by their nutritional composition. However, soybean production generates substantial vegetative biomass, which is often underutilized despite its potential as a renewable resource for green biorefineries. There is a large amount of green biomass of soybean, harvested several times in a growing season. Soybean produces substantial vegetative biomass, with yields influenced by variety, environment and harvest stage. Field studies have reported fresh biomass yields of up to 26.9 t ha^−1^ and dry matter yields of 8.9 t ha^−1^, demonstrating its potential for green biorefinery applications. Biomass partitioning among leaves, stems and pods is strongly genotype- and development-dependent, emphasizing the importance of variety selection for vegetative biomass production [32,33]. Rogers et al. [33] showed significant variation in leaf and stem biomass among genotypes and growth stages, while a Ghanaian study reported 74.2 g plant^−1^ fresh and 23.57 g plant^−1^ dry shoot biomass at flowering in inoculated plants. Medium maturing soybean varieties have a growing period of 140 days. For green biomass production, harvesting can be done on the 50th day after sowing, indicating that two harvests can be safely done within a growing season, thereby increasing the sustainability of soybean production [34]. Green biomass has favorable nutritional parameters, making it suitable for processing in green biorefineries. Leaves can be a good source of protein, while stems with a high carbohydrate content can be a precursor for second-generation fuels [7].

Lignocellulosic biomass is available in the quantities needed to replace fossil fuels, with global production of about 220 billion tonnes per year [35]. According to Martelli-Tosi, Brazilian soybean straw yields between 104 and 130 million tonnes per year.

Martelli-Tosi et al. [36] refers to soy straw as a sustainable green polymer, which offers the potential for protein and polysaccharide production based on renewable resources. Colleti et al. [37] mention that the high protein and carbohydrate content of the residual pods from the harvested soybean make it possible to get it used as a functional ingredient to increase the dietary fiber intake of the population. For the food industry, it is highly needed that such knowledge be available to apply those achievements that provide inputs with higher value to improve performance, which is closely related to operational flexibility and product development flexibility [38].

In the present research, we aimed to extend the conventional approach of soybean production by focusing on the valorization of vegetative biomass rather than solely on seed quality and nutritional properties. This study investigated the characteristics of pressed fiber obtained from soybean green biomass through wet fractionation, with particular emphasis on the effects of variety and harvest time. Based on plant anatomical, cell wall and compositional analyses of four soybean varieties harvested at two consecutive times, our aim was to evaluate the potential of soybean pressed fiber as a valuable secondary raw material for green biorefinery applications. We hypothesized that variety characteristics and vegetation window significantly influence the composition and valorization potential of soybean pressed fiber, resulting in differences among varieties regarding their suitability for further value-added utilization. Overall, this study aimed to characterize soybean-derived pressed fiber and assess its potential within a circular economy framework as an alternative pathway for sustainable soybean production.

## 2. Results

### 2.1. Plant Anatomy of the Stem

#### 2.1.1. Stem Tissues

**Figure 1:**
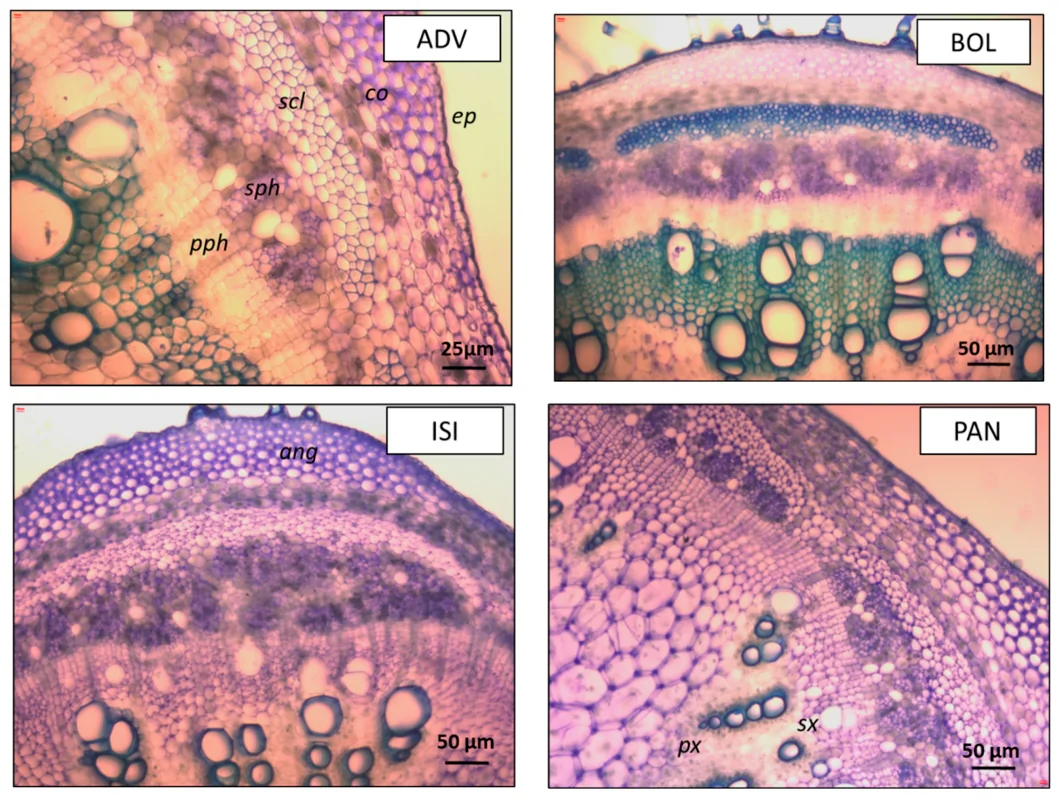
Microscopic stem cross-section of soybean (*Glycine max* L.). Cross-sections were stained with 0.2% Toluidin-blue. Co: cortex; scl: sclerenchyma; ep: epidermis; sph: secondary phloem; pph: primary phloem; ang: angular collenchima; sx: secondary xylem; px: primary xylem. ADV = Advisor; BOL = Bólyi 612; ISI = Isidor; PAN = Pannónia kincse.

The stems of soybean display the typical structure of dicotyledonous plants (Figure 1). The outermost row of cells of the rectangular stalk of the stem is formed by a single-celled epidermis of the same habit as the leaf, covered with a thin layer of cuticle. The entire length of the stem is covered with multicellular trichomes ending in a head. The hypodermis, which is composed of closely fitting cells, is divided into three sub-epidermal parts. The two to five rows of parenchymal cells forming the clorenchyma of the assimilating basal tissue all contain chloroplasts. The pith consists of mainly parenchymatous cells. A sclerenchyma cap with 5–8 cell rows is placed around the vascular bundles with a strengthening function. The thickness of the basal cells increases gradually towards the central part of the stem. Between the basal tissue cells of the stele (central cylinder), the collateral open vascular bundles are localized, composed of phloem on the outside and xylem (wood) on the inside.

#### 2.1.2. Stem Diameter, Pith Diameter and Thickness of Cortex

One-way ANOVA indicated that stem diameter did not differ significantly among the four soybean varieties (F = 2.09, *p* = 0.111). Mean stem diameter ranged from 4548.09 ± 420.9 μm in Isidor to 4920.95 ± 380.4 μm in Advisor. Tukey’s HSD post hoc test detected no significant pairwise differences among the varieties, and all varieties belonged to the same homogeneous group. Although Advisor exhibited the highest mean stem diameter, the observed variation among varieties was insufficient to demonstrate statistically significant varietal differences. The estimated effect size (η^2^ ≈ 0.09) suggested only a small to moderate influence of variety on stem diameter.

One-way ANOVA revealed no significant differences in pith diameter among the four soybean varieties (F = 2.29, *p* = 0.087). The highest mean pith diameter was recorded in Advisor (4515.10 ± 569.43 μm), whereas Isidor exhibited the lowest value (4102.73 ± 333.28 μm). Tukey’s HSD post hoc analysis did not identify any significant pairwise differences, and all varieties were assigned to the same homogeneous group. Although numerical differences among varieties were observed, these variations were not statistically significant, indicating that variety had only a limited influence on pith diameter under the experimental conditions.

The thickness of the primary cortex differed significantly among the soybean varieties (one-way ANOVA, F = 3.90, *p* = 0.013). Advisor exhibited the greatest cortex thickness (124.44 ± 16.05 μm), whereas Bólyi 612 had the lowest mean value (110.19 ± 7.20 μm). Tukey’s HSD test showed that Advisor had a significantly thicker primary cortex than Bólyi 612 (*p* = 0.0059), while Pannónia and Isidor did not differ significantly from either extreme and formed an intermediate homogeneous group. The estimated effect size (η^2^ ≈ 0.16) indicated a moderate influence of variety on primary cortex development, suggesting that varietal differences may contribute to structural variation in stem anatomy (Table 1).

#### 2.1.3. Thickness of Stem Epidermis and Cuticle

One-way ANOVA revealed significant differences in epidermis thickness among the soybean varieties. Tukey’s HSD post hoc test showed that Pannónia had a significantly thicker epidermis than both Advisor (*p* = 0.0034) and Bólyi 612 (*p* = 0.0002). In addition, Isidor exhibited significantly greater epidermis thickness than Bólyi 612 (*p* = 0.0245). No significant differences were detected between Pannónia and Isidor, Isidor and Advisor, or Advisor and Bólyi 612. These findings indicate variety-dependent variation in epidermal development, with Pannónia exhibiting the greatest epidermis thickness among the evaluated soybean varieties.

**Figure 2:**
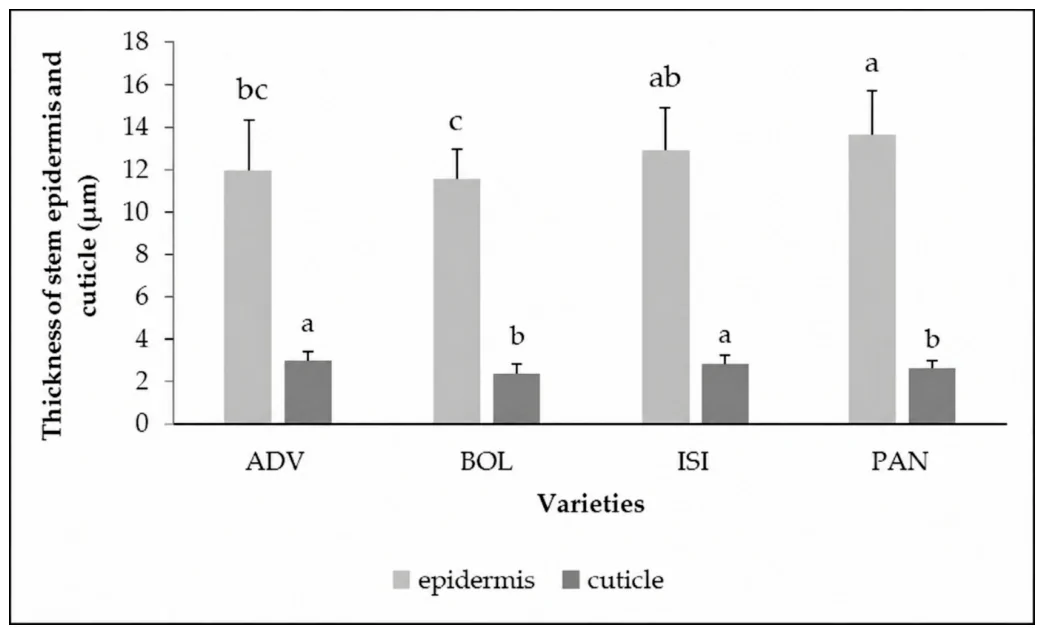
Mean (±SD) values of stem anatomical traits measured in four soybean varieties (Bólyi 612, Pannónia, Isidor and Advisor). Different lowercase letters above the bars indicate significant differences among varieties according to Tukey’s honestly significant difference (HSD) post hoc test following one-way ANOVA (*p* < 0.05). (*n* = 3); ADV = Advisor; BOL = Bólyi 612; ISI = Isidor; PAN = Pannónia kincse.

One-way ANOVA revealed significant differences in cuticle thickness among the soybean varieties. Tukey’s HSD post hoc test showed that Advisor and Isidor exhibited significantly greater cuticle thickness than Pannónia and Bólyi 612 (*p* < 0.001 for all significant comparisons). No significant differences were detected between Advisor and Isidor (*p* = 0.974) or between Pannónia and Bólyi 612 (*p* = 0.634). Accordingly, the soybean varieties were classified into two homogeneous groups: Advisor and Isidor (group a) and Pannónia and Bólyi 612 (group b). These results indicate clear variety-dependent variation in cuticle development, with Advisor and Isidor possessing a significantly thicker protective cuticle than the other two varieties (Figure 2).

### 2.2. Plant Anatomical Examination of the Leaf

#### 2.2.1. Description of the Leaf Tissue

**Figure 3:**
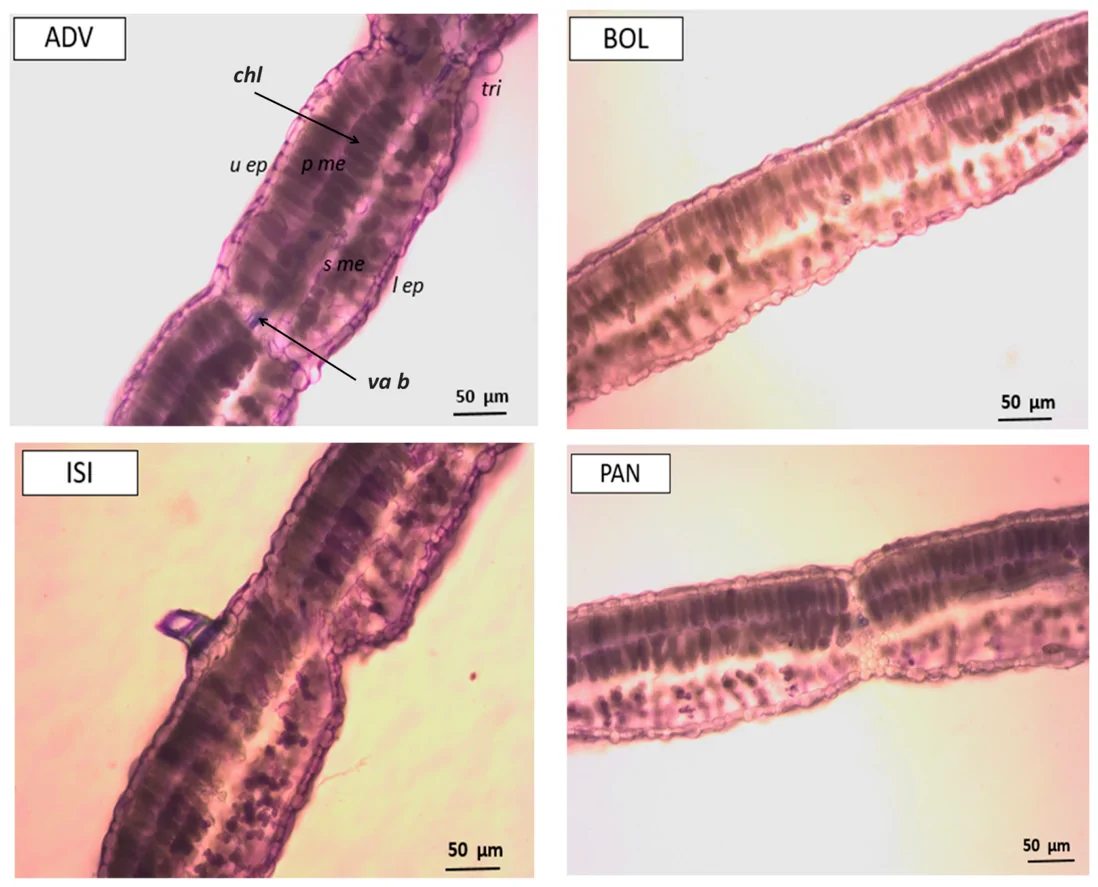
Cross-sections of soybean leaves. Cross-sections were stained with 0.2% Toluidin-blue. U ep: upper epidermis; l ep: lower epidermis; p me: palisade mesophyll; s me: spongy mesophyll; tri: trichome; chl: chlorophylls; va b: vascular bundles. ADV = Advisor; BOL = Bólyi 612; ISI = Isidor; PAN = Pannónia kincse.

On the upper and lower sides of the leaf, an epidermis of cell row thickness with a thin cuticle layer was observed. The number of cell layers forming the leaves is 7–8 layers in the leaf plate. The heterogeneous mesophyll of the leaf consists of five to six inner cell layers. The two layers of palisade parenchyma cells under the adaxial epidermis all contain chloroplasts, which are evidence of photosynthetic activity of the cells. In the leaf, water and nutrients are transported through collateral closed vessel rays (which do not contain the cambium responsible for thickening). Vascular bundles running in the leaves are localized between the spongy and palisade parenchyma. As it is typical for C3-masse plants, a sheath of one or two layers of parenchyma is observed around the rachis. The number and arrangement of the accessory vascular bundles can be considered as a fundamental difference between the varieties studied, with some leaves showing a complete absence of these bundles. A further difference is the number of cell rows in the angular collenchyma, which has a structural function, consisting of 6–8 rows, and which is responsible for the opening of the firming basal tissue. The spongy mesophyll comprises two or three cell layers and contains fewer chloroplasts than palisade cells (Figure 3).

#### 2.2.2. Thickness of Leaf Epidermis

One-way analysis of variance revealed no significant effect of soybean variety on lower epidermis thickness (F = 0.06, *p* = 0.981). The estimated effect size was negligible (η^2^ = 0.001), indicating that variety accounted for only a very small proportion of the total variability observed in this anatomical trait.

Mean lower epidermis thickness ranged from 13.23 ± 1.95 µm in Bólyi 612 to 13.41 ± 1.52 µm in Isidor. Pannónia (13.34 ± 2.06 µm) and Advisor (13.30 ± 1.55 µm) showed comparable values. Tukey’s HSD multiple comparison test confirmed the absence of significant pairwise differences among varieties, and all four varieties were assigned to the same homogeneous statistical group (group “a”).

**Figure 4:**
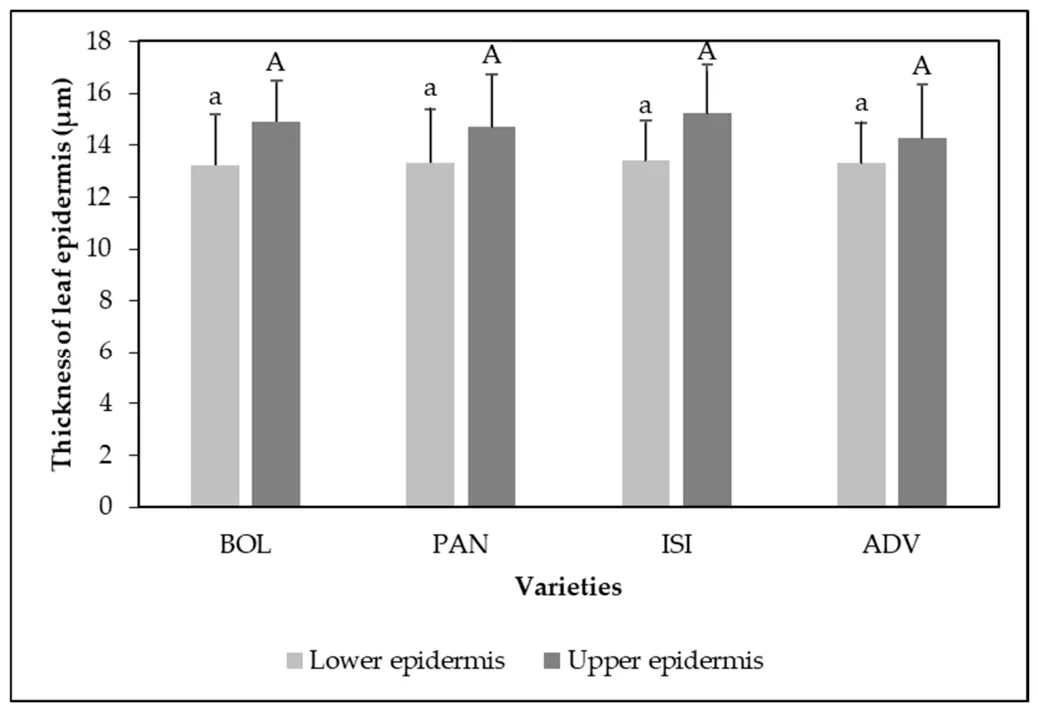
Leaf lower epidermis and upper epidermis of soybean varieties tested. Data are presented as mean ± standard deviation (SD). Different letters indicate significant differences according to one-way ANOVA followed by Tukey’s HSD test (*p* < 0.05). (*n* = 3); ADV = Advisor; BOL = Bólyi 612; ISI = Isidor; PAN = Pannónia kincse.

No significant differences were detected among the four soybean varieties regarding upper epidermis thickness according to the one-way ANOVA (F = 1.62, *p* = 0.189). The estimated effect size was small (η^2^ ≈ 0.04), indicating that variety explained only a minor proportion of the total variation. Tukey’s HSD multiple comparison test confirmed that none of the varieties differed significantly from each other, and all varieties belonged to the same homogeneous statistical group (Figure 4).

These findings indicate that soybean varieties had no significant influence on upper epidermis thickness under the experimental conditions, suggesting that this anatomical trait remained relatively stable among the investigated varieties.

#### 2.2.3. Thickness of Leaf Blade

Leaf blade thickness differed significantly among the four soybean varieties according to one-way ANOVA (F = 12.65, *p* < 0.001), indicating a strong varietal effect on leaf anatomical characteristics. The estimated effect size (η^2^ ≈ 0.22) suggested that variety explained approximately 22% of the total variation in leaf blade thickness.

**Figure 5:**
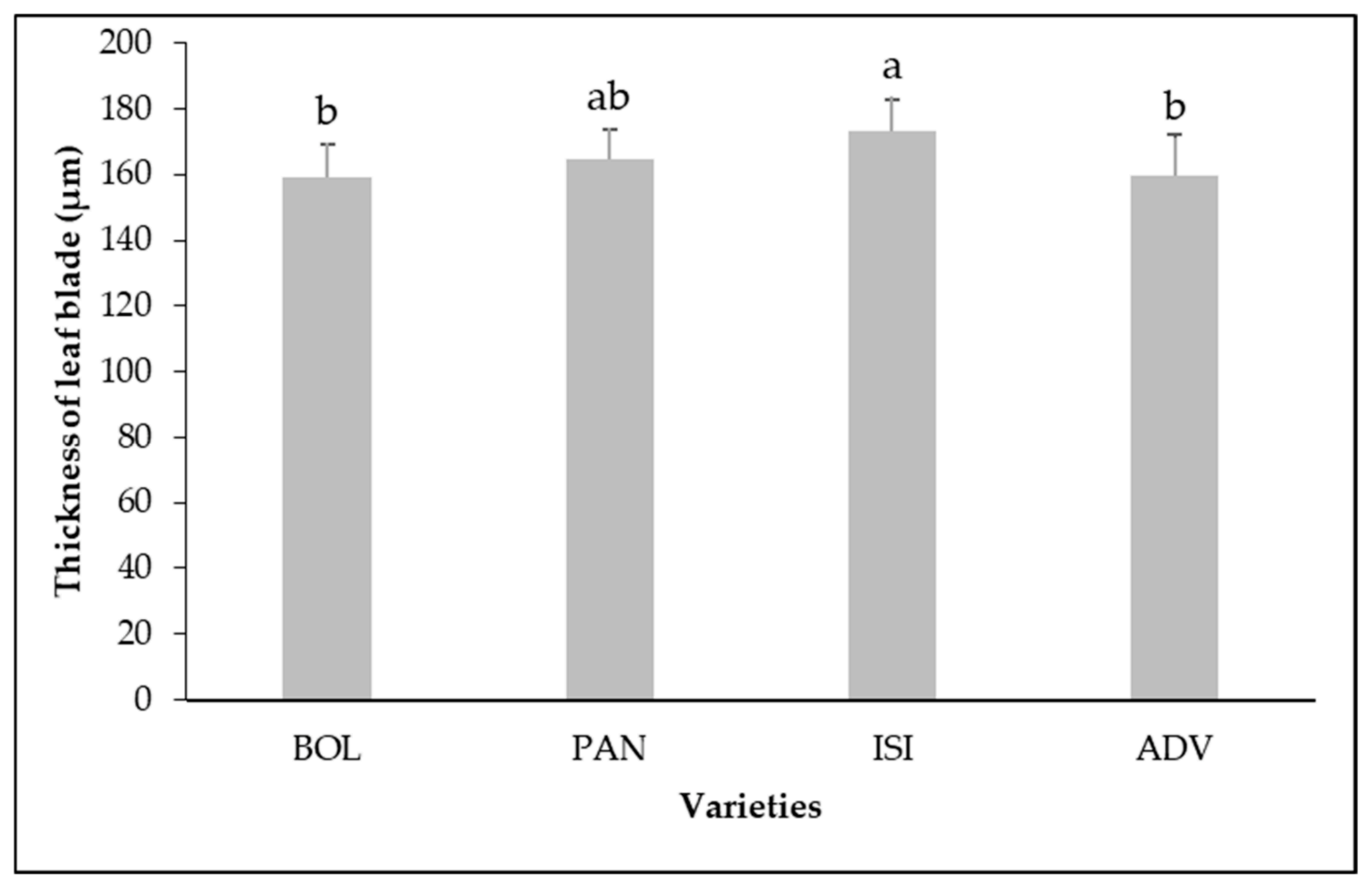
Leaf blade thickness of soybean varieties tested. Data are presented as mean ± standard deviation (SD). Different letters indicate significant differences according to one-way ANOVA followed by Tukey’s HSD test (*p* < 0.05). (*n* = 3); ADV = Advisor; BOL = Bólyi 612; ISI = Isidor; PAN = Pannónia kincse.

The highest mean leaf blade thickness was recorded for the variety Isidor (175.16 ± 10.34), whereas the lowest value was observed for Bólyi 612 (156.82 ± 13.94). Pannónia (165.45 ± 9.25) and Advisor (161.92 ± 16.62) exhibited intermediate values. Tukey’s HSD test showed that Isidor formed the highest statistical group and differed significantly from Bólyi 612, while Pannónia occupied an intermediate position, and Advisor did not differ significantly from the adjacent groups (Figure 5).

### 2.3. Biochemical Analyses

#### 2.3.1. Lignocellulosic Components

The glucan content of soybean varieties differed significantly among varieties and between the two vegetation windows. Two-way ANOVA revealed a significant effect of variety (F(3,40) = 3.773, *p* = 0.0178, η^2^p = 0.221) and vegetation window (F(1,40) = 8.868, *p* = 0.0049, η^2^p = 0.181), whereas the variety × vegetation window interaction was not significant (F(3,40) = 1.251, *p* = 0.304).

The highest glucan concentration was measured in the ADV variety during the first vegetation window (32.09%), followed by ISI during the same vegetation window (31.74%). The lowest values were observed in ISI and BOL during the second vegetation window (27.54%). Tukey’s HSD test confirmed significant differences among treatment combinations, indicated by different letter groups.

The structural carbohydrate composition of soybean biomass differed between the two vegetation windows. Xylan, arabinan and Klason lignin contents showed significant differences between vegetation windows, while variety effects and variety × vegetation window interactions were not significant.

**Figure 6:**
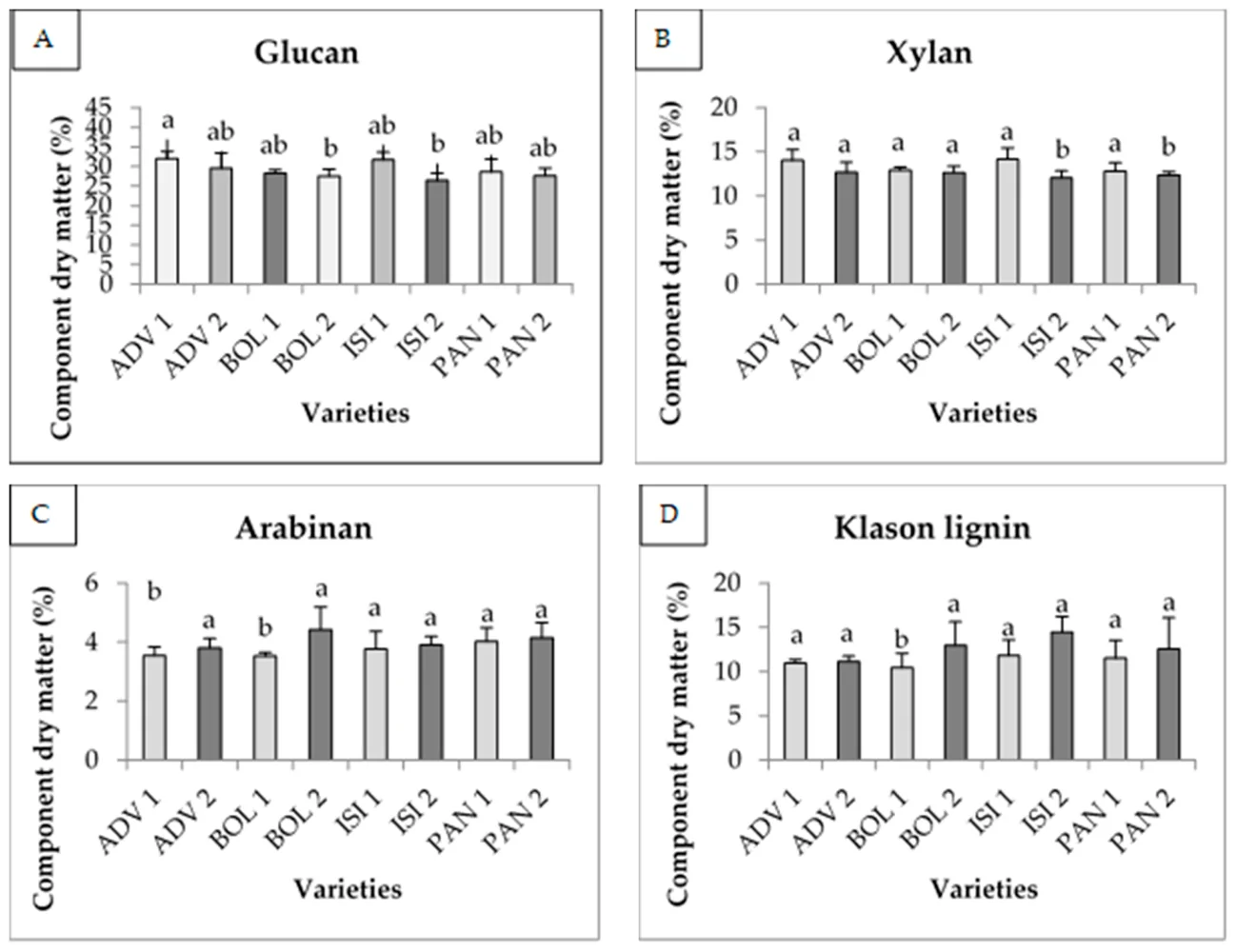
Effect of soybean variety and harvest time on the measured glucan (**A**), xylan (**B**), arabinan (**C**) and Klason lignin (**D**) concentrations. Values represent the mean ± standard deviation (SD) of the biological replicates. Different lowercase letters indicate statistically significant differences among treatments according to Tukey’s HSD test (*p* < 0.05). The effects of variety, harvest time, and their interaction were evaluated using two-way ANOVA. (total *n* = 48) *n* = 3/treatment; ADV = Advisor; BOL = Bólyi 612; ISI = Isidor; PAN = Pannónia kincse.

Xylan concentration differed significantly between the two vegetation windows (F(1,40) = 14.965, *p* < 0.001, η^2^p = 0.272), with higher values observed during the first vegetation window. Arabinan content also differed significantly between vegetation windows (F(1,40) = 7.100, *p* = 0.011, η^2^p = 0.151). Similarly, Klason lignin content showed significant variation between vegetation windows (F(1,37) = 6.950, *p* = 0.012, η^2^p = 0.158). The lack of significant variety × vegetation window interactions indicates that no statistically significant evidence was found for differential responses among the examined soybean varieties across the two vegetation windows (Figure 6).

#### 2.3.2. Photosynthetic Pigments

The two-way ANOVA indicated that soybean variety had no significant effect on chlorophyll a concentration (F = 1.80, *p* = 0.187). Likewise, vegetation window did not significantly influence chlorophyll a content (F = 0.27, *p* = 0.611), and no significant interaction was detected between variety and vegetation window (F = 0.29, *p* = 0.832). These results indicate that chlorophyll a concentration remained relatively stable among the investigated soybean varieties and between the two vegetation windows. The effect sizes were small, indicating that only a minor proportion of the observed variability could be attributed to the investigated experimental factors.

The two-way ANOVA revealed that soybean variety had no significant effect on chlorophyll b concentration (F = 1.59, *p* = 0.223). Similarly, vegetation window did not significantly influence chlorophyll b content (F = 0.41, *p* = 0.529), and no significant interaction between variety and vegetation window was observed (F = 0.37, *p* = 0.777). Overall, chlorophyll b concentration remained relatively stable across the investigated soybean varieties and between the two vegetation windows. The estimated effect sizes (partial η^2^) were low, indicating that the examined experimental factors accounted for only a small proportion of the total variation in chlorophyll b concentration.

The two-way ANOVA demonstrated that soybean variety had no statistically significant effect on total carotenoid concentration (F = 2.14, *p* = 0.133). Likewise, vegetation window did not significantly influence total carotenoid content (F = 0.56, *p* = 0.465), and no significant interaction between variety and vegetation window was detected (F = 0.48, *p* = 0.699). Although varietal differences accounted for a slightly greater proportion of the observed variation than vegetation window, the corresponding effect size remained small and did not reach statistical significance. Overall, total carotenoid concentration was relatively stable among the investigated soybean varieties and between the two vegetation windows, indicating that neither variety nor vegetation window showed a statistically significant effect on pigment accumulation under the conditions of the present experiment.

**Figure 7:**
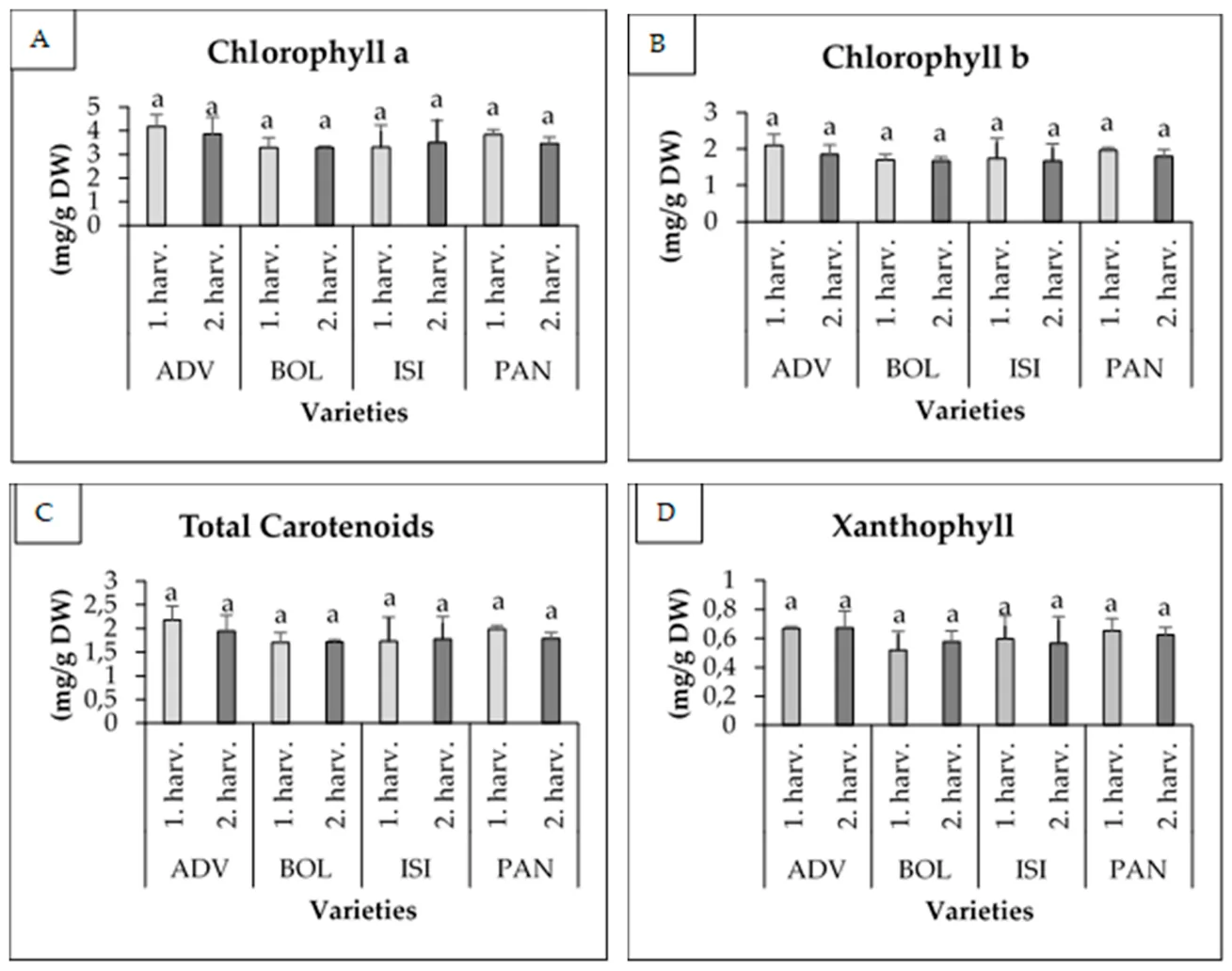
The photosynthetic pigment content of pressed fiber of soybean varieties: (**A**) chlorophyll a; (**B**) chlorophyll b; (**C**) total carotenoids; and (**D**) xanthophyll, as a function of harvest time. Values represent the mean ± standard deviation (SD) of the biological replicates. Different lowercase letters indicate statistically significant differences among treatments according to Tukey’s HSD test (*p* < 0.05). The effects of variety, harvest time, and their interaction were evaluated using two-way ANOVA. (total *n* = 24) *n* = 3/treatment; 1. harv. = first harvest of soybean, 2. harv. = second harvest of soybean. DW = Dry weight; ADV = Advisor; BOL = Bólyi 612; ISI = Isidor; PAN = Pannónia kincse.

The two-way ANOVA showed that soybean variety had no significant effect on xanthophyll concentration (F = 1.47, *p* = 0.257). Similarly, vegetation window did not significantly affect xanthophyll content (F = 0.32, *p* = 0.579), and the interaction between variety and vegetation window was also not significant (F = 0.41, *p* = 0.748). Partial eta-squared values indicated only small effect sizes for all investigated factors, suggesting that the observed variation in xanthophyll concentration was largely independent of soybean variety and vegetation window. Overall, xanthophyll content remained stable among the four soybean varieties and between the two vegetation windows under the experimental conditions (Figure 7).

#### 2.3.3. Mineral Elements

The micro- and macro-element content is shown in Table 2. During the first vegetation window, soybean variety significantly affected the concentrations of Ca, Cu, Mn, and Mo in the analyzed plant material (one-way ANOVA, α = 0.05). A significant varietal effect was observed for Ca concentration (F_3,8_ = 4.78, *p* = 0.034, η^2^ = 0.642), indicating a relatively large effect of variety. Tukey’s HSD post hoc analysis showed that BOL had a significantly higher Ca concentration than ADV, whereas the other pairwise comparisons were not significant. Copper concentration also differed significantly among varieties (F_3,8_ = 6.25, *p* = 0.017, η^2^ = 0.701). According to Tukey’s HSD test, BOL exhibited a significantly higher Cu concentration than PAN, while the remaining pairwise comparisons were not significant. A significant varietal effect was likewise detected for Mn concentration (F_3,8_ = 4.96, *p* = 0.031, η^2^ = 0.650). BOL showed significantly higher Mn concentration than PAN, whereas ADV and ISI did not differ significantly from either variety. The strongest varietal effect was observed for Mo (F_3,8_ = 13.07, *p* = 0.0019, η^2^ = 0.831). Tukey’s HSD analysis demonstrated that PAN had significantly lower Mo concentration than ADV, BOL, and ISI, indicating a pronounced varietal difference in Mo concentration during the first vegetation window. For B, Ba, Fe, K, Mg, Na, P, S, Sr, and Zn, no statistically significant differences among soybean varieties were detected (*p* > 0.05). Although relatively large effect sizes were observed for some elements, including Mg and Sr, these differences were not statistically significant and should therefore be interpreted cautiously.

The micro- and macro-element content is shown in Table 3. At the second vegetation window, soybean variety significantly affected the concentrations of K, Mn, Mo, and P in the analyzed plant material, whereas no significant varietal differences were detected for B, Ba, Ca, Cu, Fe, Mg, Na, S, Sr, or Zn (one-way ANOVA, α = 0.05). The strongest varietal effect was observed for K concentration (F_3,8_ = 10.36, *p* = 0.004, η^2^ = 0.795). Tukey’s HSD test showed that K concentration was significantly higher in ADV than in BOL (*p* = 0.003), and PAN also had a significantly higher K concentration than BOL (*p* = 0.017). ISI showed an intermediate response and did not differ significantly from the other varieties. Manganese concentration was also significantly affected by variety (F_3,8_ = 6.76, *p* = 0.014, η^2^ = 0.717). BOL and ISI exhibited significantly higher Mn concentrations than PAN (*p* = 0.017 and *p* = 0.021, respectively), while ADV showed an intermediate response. A significant varietal effect was observed for Mo concentration (F_3,8_ = 6.95, *p* = 0.013, η^2^ = 0.723). ADV exhibited a significantly higher Mo concentration than both BOL (*p* = 0.018) and PAN (*p* = 0.034), whereas ISI showed an intermediate response and did not differ significantly from either group. Phosphorus concentration also differed significantly among varieties (F_3,8_ = 6.17, *p* = 0.018, η^2^ = 0.698). PAN showed significantly higher P concentrations than BOL (*p* = 0.020) and ISI (*p* = 0.034), while ADV displayed an intermediate response. No statistically significant varietal effects were observed for B, Ba, Ca, Cu, Fe, Mg, Na, S, Sr, or Zn (*p* > 0.05). Although some of these elements showed moderate effect sizes, the observed differences did not reach the predefined significance level. Overall, the results indicate that varietal differences in elemental composition within the second vegetation window were element-specific, with particularly pronounced effects observed for K, Mn, Mo, and P.

## 3. Discussion

Innovative processing of soybean green biomass, which can be produced in considerable quantities, may contribute to the development of green biorefinery systems based on circular biomass utilization. In the present study, we investigated the characteristics and potential utilization of pressed fiber obtained by wet fractionation of soybean green biomass. Four soybean varieties (Advisor, Bólyi 612, Isidor, and Pannónia kincse) were evaluated across two distinct vegetation windows in 2021, with emphasis on plant anatomical characteristics, cell-wall polymer composition, photosynthetic pigments, and elemental concentrations.

The anatomical analysis revealed significant varietal differences in several measured parameters, indicating variation in the structural characteristics of the vegetative organs. Leaf blade thickness was highest in Isidor (173.15 µm) and lowest in Bólyi 612 (159.29 µm), which is consistent with previous observations in soybean [39,40,41,42]. Differences were also detected in stem anatomical parameters, including stem diameter and tissue thickness. These results demonstrate that soybean varieties differ in their anatomical structure, which may influence the composition and physical characteristics of the biomass obtained for further processing. However, the present measurements do not allow direct conclusions regarding stress tolerance, lodging resistance, or mechanical harvesting performance. Such relationships should be evaluated in future studies by combining anatomical measurements with direct mechanical and field-performance assessments.

The analysis of cell-wall components showed clear variation between the investigated vegetation windows. Klason lignin content was higher during the second vegetation window across the varieties, with the highest values observed in Isidor (11.82 and 14.48% in the first and second vegetation windows, respectively). These results are in agreement with previous observations of changes in soybean cell-wall composition during vegetation [43,44,45]. The measured glycosyl residues, including glucan, xylan, and arabinan, also varied according to variety and vegetation window, indicating that the composition of the pressed fiber varied across varieties and sampling periods. These differences are relevant for assessing the potential of soybean pressed fiber as a lignocellulosic secondary biomass resource. However, because the two vegetation windows represented separate cultivation periods occurring at different times of the growing season, the observed differences cannot be exclusively attributed to developmental stage or sampling time and may also reflect differences in environmental conditions.

Photosynthetic pigments were also detected in the pressed fiber, confirming the retention of chlorophyll and carotenoid compounds after wet fractionation. In contrast to the cell-wall components, pigment composition showed relatively limited variation among the investigated varieties and vegetation windows. The two-way ANOVA confirmed that neither variety nor vegetation window had a significant effect on chlorophyll a, chlorophyll b, total carotenoid, or xanthophyll concentrations, and no significant variety × vegetation window interactions were detected. This suggests that the pressed fiber retained a relatively consistent pigment fraction under the conditions of the present experiment. The nutritional or functional significance of these compounds, however, requires further characterization before specific food or feed applications can be proposed.

Elemental analysis further demonstrated that soybean pressed fiber contains both macro- and microelements. Variety-dependent differences were observed within each vegetation window, although the pattern was element-specific. During the first vegetation window, significant differences among varieties were detected for Ca, Cu, Mn, and Mo, whereas K, Mn, Mo, and P differed significantly among varieties during the second vegetation window. In particular, Mn and Mo showed significant variety-related variation in both vegetation windows. Because these elemental data were analyzed separately within each vegetation window using one-way ANOVA, the present analysis does not directly test the main effect of vegetation window or the variety × vegetation window interaction. Therefore, the differences observed between the two vegetation windows should be cautiously interpreted rather than as evidence of an independent vegetation-window effect on elemental composition. These findings extend the characterization of soybean green biomass beyond its structural components and support further investigation of its potential as a source of multiple biomass fractions within integrated biorefinery systems.

Overall, the present results provide a baseline characterization of soybean pressed fiber obtained from four varieties and two distinct vegetation windows. As this study was conducted at one site during a single growing season, the observed differences between vegetation windows should be interpreted within the specific experimental conditions. In particular, the two vegetation windows occurred during different calendar periods and were therefore associated with potentially different temperatures, precipitation, radiation, and other environmental conditions. Consequently, the observed differences cannot be interpreted as effects of plant developmental stage or harvest timing alone, and the present study does not provide evidence for a generally optimal harvest stage. Further multi-year and multi-environment studies, including precisely defined developmental stages and direct measurements of mechanical properties, stress responses, and potential functional applications, are needed to validate and extend the present findings.

## 4. Materials and Methods

### 4.1. Source of Soybean Biomass

Materials used were obtained from a small plot field in the experimental site of the University of Debrecen, Debrecen, Hungary (47° 55′ N, 21 °60′ E). After the plants were collected, our work consisted of two activities. Plant anatomical studies aimed at revealing the tissue structure, while analytical measurements were performed to study the phytochemical components of the pressed fiber fraction of the varieties.

#### 4.1.1. Experimental Setup

Four soybean varieties (Advisor, Bólyi 612, Pannónia Kincse, Isidor) grown in Hungary were set up in 3 replications. Two early and two medium maturing varieties were deliberately included in this study. Advisor: early-maturing variety, growing time 128–132 days, growth type semi-determinate, weight per thousand seeds 190–220 g, recommended row spacing 24–45 cm, sowing time mid to late April. Bólyi 612: early-maturing variety, maturing in 130–140 days, semi-determinate growth type, 180–210 g per thousand seeds, recommended row spacing 24–76 cm, sowing from mid-April to mid-May. Isidor: medium-maturing variety, growing time 130–145 days, growth type determinate, weight per thousand seeds 200–220 g, recommended row spacing 45 cm, sowing from 10 April to 30 April. Pannónia kincse: medium-maturing variety, maturing in 135–145 days, growth type interdeterminate, weight per thousand seeds 180–190 g, recommended row spacing 24–76 cm, sowing mid-April to early May.

During the soybean experiment, a randomized complete block design was applied for the small-plot trials (Table 4). The plot size was 4.52 m^2^, and the sowing arrangement was 24 × 10 cm. Unlike the row spacing commonly applied in the literature, a wider row distance of 24 cm was selected, as the primary objective of our experiment was not seed production but the investigation of green biomass characteristics. Based on this objective, the experiment was established twice consecutively within the same growing season, allowing the evaluation of soybean biomass at two different developmental periods.

The first vegetation window lasted from 11 May 2021 until the end of June 2021, while the second vegetation window was established from 13 July 2021 until the end of August 2021. In both cases, biomass collection was targeted at the green bud stage, based on the intended developmental stage for biomass sampling. The two vegetation windows therefore represented separate cultivation periods occurring during different calendar periods and under potentially different environmental conditions.

During autumn soil preparation, 150 kg ha^−1^ of a complex NPK fertilizer (7-21-21) was applied as a basal nutrient supply. Mechanical weed control was performed twice during the growing season until canopy closure, ensuring optimal plant development and minimizing weed competition. The soybean stands did not receive any fungicide or insecticide treatments during the vegetation period. No irrigation was applied during the entire growing period; therefore, the soybean plants were grown under natural precipitation conditions.

Following harvest, the fresh biomass samples were immediately transported to the laboratory, where they were processed without delay, and pressed fiber samples were prepared according to the methodology described below. For the preparation of histological sections, the plant samples were placed in a preservation solution, the details of which are also described in the following sections.

In the figures and tables of the thesis, the following abbreviations were used for the individual soybean varieties: Es Advisor (ADV), Bólyi 612 (BOL), Isidor (ISI), and Pannónia kincse (PAN). The numbers 1 and 2 following the variety names indicate the respective harvest dates.

#### 4.1.2. Climatic Condition

**Figure 8:**
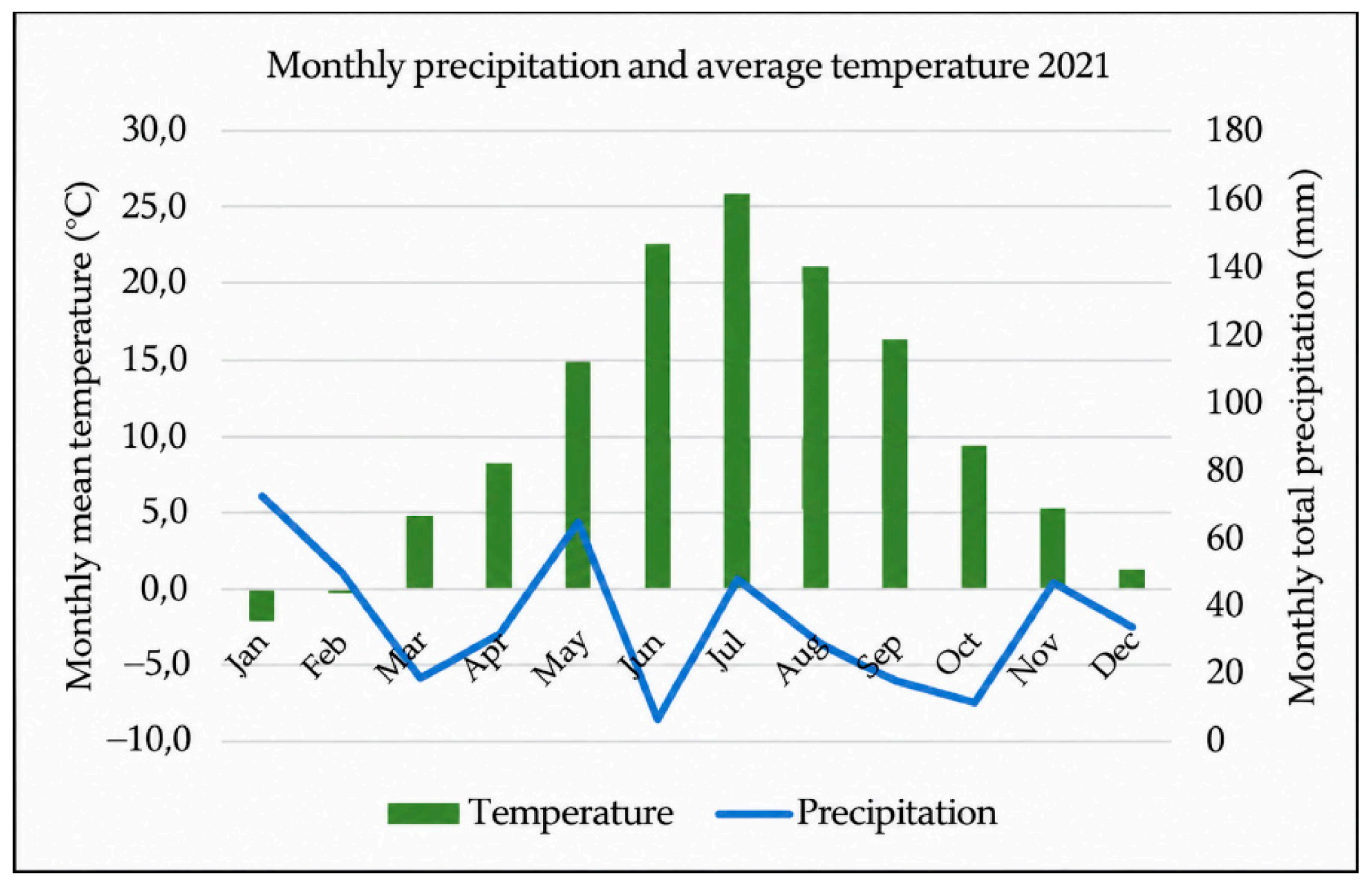
Temperature and precipitation conditions during the experimental year.

Temperature conditions were generally favorable for soybean development, with average temperatures ranging from 14.9 °C in May to 25.9 °C in July, providing suitable thermal conditions for vegetative growth and reproductive development. However, precipitation distribution was highly uneven, with only 197.6 mm rainfall recorded during the April–September growing period. The most critical water deficit occurred in June, when only 6 mm precipitation was recorded, coinciding with intensive vegetative development. Therefore, although thermal conditions were favorable, water availability represented the major environmental factor limiting soybean production potential during the growing season (Figure 8).

#### 4.1.3. Soil Characteristics of the Experimental Site

The experimental soil was characterized by a neutral pH (pH_KCl = 6.8), providing favorable conditions for nutrient availability and microbial activity. The humus content (2.57%) indicated a medium level of organic matter, while the Arany plasticity index (KA = 42) classified the soil as a loam to clay loam with moderate water-holding capacity. The AL-extractable phosphorus and potassium contents were 100 and 165 mg kg^−1^, respectively, suggesting an adequate phosphorus supply and a moderate potassium status for crop production. Overall, the soil properties provided suitable conditions for evaluating the effects of the experimental treatments under field conditions (Table 5).

### 4.2. Morphological and Anatomical Studies

#### 4.2.1. Sample Preparation

Leaf and stem anatomical characteristics were determined by observing the leaf and stem samples prepared. The vegetative organs were sampled on day 47 after sowing. Stem and leaf samples were collected from three randomly selected individuals per replicate. According to Lersten–Carlson [46], terminal leaflets of trifoliate leaves from the fifth node of plants were collected, including the main vein at the same phenological stage (in the reproductive stage of ontogenesis, the initial flowering (R1) stage); at this time, all plants in the plot in the green bud stage were collected based on Horváth et al. [34], while internodes (stem segments) between the fourth and fifth node of stems were collected. Samples were fixed in Strasbourger–Flemming solution (glycerol:water:alcohol 1:1:1) in individually coded sample bottles (two weeks), which promoted softening.

#### 4.2.2. Anatomical Analysis

The samples were cut by hand using sharp blades. The sampling hierarchy was as follows: plot → plant → section → image/measurement, with plots considered the independent biological replicates. The experimental design included two independent experimental factors, namely soybean variety and vegetation window. The individual field plot was considered the experimental unit and biological replicate. For each variety × vegetation window combination, three independent plots were evaluated (*n* = 3 biological replicates). From each plot, five plants were randomly selected. From each individual plant, five stem cross-sections and five leaf cross-sections were prepared. Five measurements were performed for each anatomical parameter on each individual cross-section. Thus, the anatomical measurements represented a hierarchical sampling structure consisting of three independent plots, five plants per plot, five sections per plant, and five measurements per section. The plot was considered the independent biological replicate, whereas plants, cross-sections, and repeated measurements within each plot represented subsamples of the corresponding biological replicate. Consequently, the multiple measurements obtained from the same plot were not considered independent biological replicates in the statistical analysis. The collected samples were placed in a 1:1 mixture of sodium hypochlorite and distilled water. They were stained with Toluidin-blue. Observations were made on the morphological characteristics of individual leaves of soybean. For soybean leaf, three parts (leaf blade thickness, lower epidermis thickness, upper epidermis thickness) were quantified and for stem, five additional parameters were quantified (total stem diameter, epidermis thickness, stele diameter, primary cortex thickness, cuticle thickness).

For the measurements, a Zeiss Axioskop 2+ light microscope with different magnification objectives (2.5×, 4×, 10×, 20×, 40×) was used. All sections were digitized and the investigated parts were measured on the photographs using the Scope Photo program. Depending on the quality of the digitised cross-section images, 1–2 measurments per section were conducted.

### 4.3. Pressed Fiber Production by Wet Fractionation

The quantity of the biomass samples collected from each variety was 1 kg per plot. The plant was cut above 15 cm from the bottom. From the collected green biomass, the fiber fraction was prepared by wet fractionation using a twin screw press juicer (Angel Juicer 5500, Angel Ltd., Praha, Czech Republic). The fiber fraction was frozen, lyophilized and then chopped using a coffee grinder and used in this form for our measurements, which we will refer to as the pressed fiber sample. The sampling hierarchy was as follows: plot → fiber sample → measurements, with the plot serving as the independent biological replicate and repeated measurements representing analytical replicates.

#### 4.3.1. Determination of Recoverable Sugars

**Figure 9:**
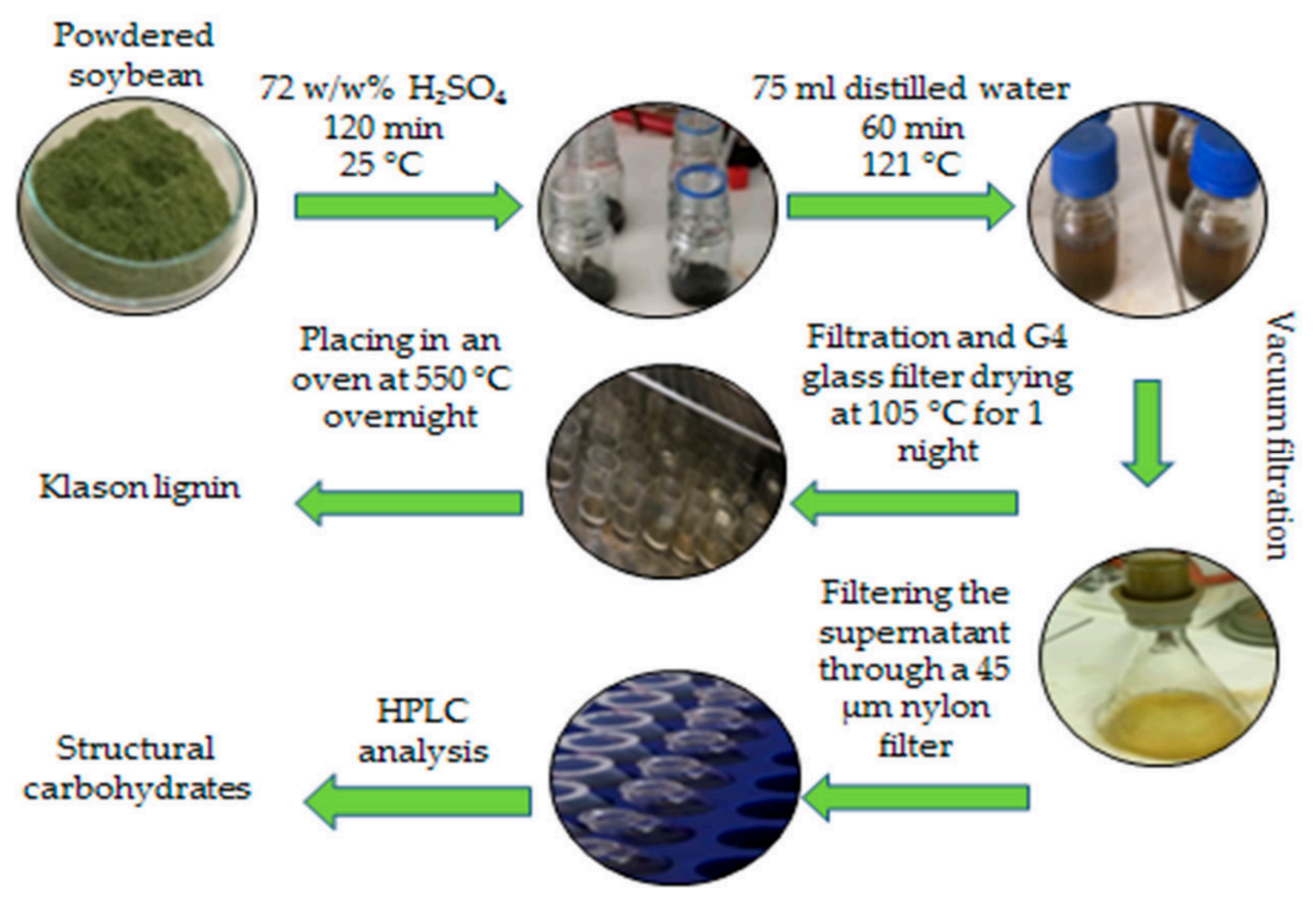
Determination of Klason lignin and structural carbohydrates.

From the aforementioned lyophilized powdered pressed fiber samples, 0.5 g were measured, and the cell wall constituents were determined using a modified NREL method (extended autoclave hydrolysis time of 2 h at 121 °C) based on Sluiter [47]. Monosaccharides (glucose, xylose, arabinose) were determined by high-performance liquid chromatography (HPLC) at 65 °C. Detector: Shimazu RID-10 A; Column: BioRad (Hercules, CA, USA) Aminex HPX-87H (300 × 7.8); Eluent: 5 mmol L^−1^ sulphuric acid; Flow rate: 0.5 mL min^−1^. Briefly, 0.5 g of lyophilized sample was weighed in a laboratory flask and then placed in 72% sulphuric acid for two hours, with stirring every 30 min. It was then placed in an autoclave at 121 °C for two hours. Next, the sample was filtered through a G4 glass filter, and the supernatant was filtered through a 45 μm nylon filter. This sample was analyzed by HPLC. All sample preparation, processing, and data evaluation were performed by the authors, while the instrumental HPLC measurements were carried out by an external analytical laboratory. The process is shown in Figure 9. During the protocol implementation, the glucan (glucan includes all polysaccharides consisting of glucose monomers), xylan, and arabinan contents were calculated using the appropriate formulae:

Glucan (component dry matter %) = [((C_glucose_ × dilution × V_supernatant_)/hydration factor)]/m × DM

Xylan (component dry matter %) = [((C_xylose_ × dilution × V_supernatant_)/hydration factor)]/m × DM

Arabinan (component dry matter %) = [((C_arabinose_ × dilution × V_supernatant_)/hydration factor)]/m × DM

C: concentration of sugar monomer;

V: 0.0775 L;

Hydration factor: glucose: 1.136; xylose, arabinose: 1.111;

m: weight of sampled pressed fiber sample;

DM: dry matter.

#### 4.3.2. Determination of Klason Lignin

G4 glass filters were placed in a desiccator and weighed after 30 min. Using the modified NREL-method, after filtration and washing, the water-insoluble part remains in the glass filter. The glass filter was then placed in an oven at 105 °C overnight and then weighed. The glass filter was then placed in an oven at 550 °C overnight and then weighed. Finally, the Klason lignin content was determined using the appropriate formula and corrected for ash content:

Klason lignin (%) = {[(m_2_ −m_1_)−m_ash_]/m_0_} × 100

m_0_: oven-dried sample mass (g);

m_1:_ mass of the empty filter crucible/filter (g);

m_2_: mass of the filter crucible/filter containing the dried acid-insoluble residue after hydrolysis (g);

m_ash_: mass of acid-insoluble ash after combustion (550 °C) of the residue (g).

### 4.4. Determination of Micro- and Macro Element Content from Pressed Fiber Fractions

The micro- and macroelement content of the pressed fiber fraction was determined from powdered and lyophilized samples from two harvests. As a first step in the process, 1 g of representative plant sample was analyzed. A total of 10 cm^3^ HNO_3_ was added to the sample and destructed in a laboratory MIM OE 718/A block crusher for 30 min at 60 °C. In the second phase of the destoning, an additional 10 cm^3^ of 30% H_2_O_2_ was added to the cooled samples, and subsequently the samples were treated at 120 °C for 90 min. Following thermostatting, 50 cm^3^ of distilled water was added to our samples, and after filtration with MN 640W filter paper, the measurement was performed using a Thermo Electron Corporation iCAP 6300 Dual Inductively Coupled Plasma Optical Emission Spectroscope (ICP-OES) [48]. All sample preparation, processing, and data evaluation were performed by the authors, while the instrumental ICP-OES measurements were carried out by an external analytical laboratory. The following elements were determined: barium (Ba), boron (B), calcium (Ca), copper (Cu), iron (Fe), magnesium (Mg), manganese (Mn), molybdenum (Mo), phosphorus (P), potassium (K), sodium (Na), strontium (Sr), sulphur (S) and zinc (Zn).

### 4.5. Determination of Photosynthetic Pigment Content

Photosynthetic pigments were determined by the spectrophotometric method. A total of 10 mg of the powdered lyophilized pressed fiber samples was measured in an Eppendorf tube, into which 2 mL of 96% ethanol was pipetted (powdering makes the sample more permeable to the solvent, which helps to release the pigments). Subsequent to mixing using a vortex, the sample was incubated in an ultrasonic bath (instrument: USC200TH, ultrasonic frequency: 45 kHz, temperature: 25 °C) in the dark for 1 h. After incubation, the sample was centrifuged at 13,000 rpm, and the supernatant was used for subsequent measurements. The absorbances were observed at the following wavelengths: 440 nm, 480 nm, 495 nm, 649 nm, 665 nm. Instrument: Ultraspec 2100 pro (Amersham BioSciences) spectrophotometer. Subsequent to substitution into the appropriate formula, the chlorophyll a, chlorophyll b, total carotenoids and xanthophyll contents within total carotenoids were obtained [49].

**Chlorophyll a (mg g^−1^)** = [(13.7 × A_665_) − (5.76 × A_649_)]/mass × 200

**Chlorophyll b (mg g^−1^)** = [(25.8 × A_649_) − (7.6 × A_665_)]/mass × 200

**Total carotenoids (mg g^−1^)** = [(4.7 × A_440_) − (0.263_Chla + Chlb_)]/mass × 200

**Xanthophylls (lutein) (mg g^−1^)** = [(11.51 × A_480_) − (20.61 × A_495_)]/mass × 200

A: absorbance;

mass: weight of pressed fiber sample.

### 4.6. Statistical Analysis

The statistical analysis was performed using one-way analysis of variance (one-way ANOVA) to evaluate the differences among the experimental groups for each investigated parameter (leaf blade, leaf lower epidermis, leaf upper epidermis, stem diameter, stem pith diameter, thickness of stem primary cortex, stem epidermis, stem cuticle, mineral elements). The measured traits were considered as dependent variables, while the treatment groups were included as independent factors in the statistical model.

Before performing the analysis of variance, the assumptions required for one-way ANOVA were tested. The normality of data distribution was assessed using the Shapiro–Wilk test, while the homogeneity of variances was evaluated using Levene’s test. The assumptions of independence of observations, normal distribution of residuals, and homogeneity of variances were confirmed prior to ANOVA.

When significant differences were detected by ANOVA (*p* < 0.05), Tukey’s honestly significant difference (HSD) post hoc test was performed for pairwise comparisons among the treatment means. Statistical significance was accepted at a significance level of α = 0.05.

All results were expressed as mean ± standard deviation (SD). Different lowercase letters indicate statistically significant differences among treatments according to Tukey’s HSD test.

The effects of soybean variety and harvest time on the investigated parameters (glucan, xylan, arabinan, Klason lignin, photosynthetic pigments) were evaluated using a two-way analysis of variance (ANOVA). The following linear model was applied:

[Y_{ijk} = \mu + V_i + H_j + (V\times H){ij} + \varepsilon{ijk}]

where:

- (Y_{ijk}) = observed value of the measured parameter;
- (\mu) = overall mean;
- (V_i) = fixed effect of the ith soybean variety;
- (H_j) = fixed effect of the jth harvest time;
- ((V\times H)_{ij}) = interaction effect between soybean variety and harvest time;
- (\varepsilon_{ijk}) = random experimental error, assumed to be independently and normally distributed with a mean of zero and constant variance ((\varepsilon\sim N(0,\sigma^2^))).

The significance of the main effects (variety and harvest time) and their interaction was evaluated using F-tests. Prior to the ANOVA, the assumptions of normality and homogeneity of variances were verified using the Shapiro–Wilk and Levene’s tests, respectively. When significant differences were detected (*p* < 0.05), mean separation was performed using Tukey’s Honestly Significant Difference (HSD) post hoc test. Effect sizes were estimated using partial eta squared (η^2^p). Descriptive statistics were calculated for each treatment group, including the mean and standard deviation (SD). Although 95% confidence intervals (CIs) were also calculated, they are not presented in the manuscript.

The data were statistically processed using Microsoft Excel 2016 and IBM SPSS 24 statistical software.

## 5. Conclusions

The present study provides a comprehensive first characterization of soybean green biomass-derived pressed fiber as a potential secondary raw material for green biorefinery applications. Unlike conventional soybean production approaches focusing primarily on seed yield and nutritional quality, this research highlights the potential value of vegetative biomass and demonstrates variation in its anatomical, structural, and biochemical properties among soybean varieties and between two distinct vegetation windows.

The anatomical analyses revealed variety-dependent differences in several structural traits, particularly in primary cortex thickness, epidermal development, cuticle thickness, and leaf blade thickness, whereas stem diameter, pith diameter, and several epidermal characteristics remained relatively stable among varieties. These findings indicate that soybean varieties exhibit distinct anatomical characteristics, which may contribute to differences in biomass structure and potential processing properties.

The biochemical analyses demonstrated variation in the composition of soybean pressed fiber among varieties and between the two vegetation windows. Glucan content was significantly influenced by both variety and vegetation window, whereas xylan, arabinan, and Klason lignin contents showed significant differences between vegetation windows. The absence of significant variety × vegetation window interactions for these parameters indicates that the examined varieties generally showed similar patterns of variation across the two vegetation windows. However, because the two vegetation windows corresponded to different calendar periods, the observed differences cannot be exclusively attributed to plant developmental status or harvest timing. Rather, they may reflect a combined influence of plant developmental status and the environmental conditions prevailing during the respective growing periods. Therefore, the present experimental design does not allow the identification of an optimal harvest stage independently of seasonal environmental variation. In contrast, pigment-related parameters showed limited variation, suggesting relatively stable chlorophyll and carotenoid accumulation under the investigated experimental conditions.

It should be emphasized that this study represents a one-year exploratory investigation aimed at identifying variation patterns among soybean varieties and vegetation windows rather than establishing definitive recommendations regarding variety suitability or optimal harvesting strategies. The observed differences provide valuable preliminary information regarding the factors influencing soybean pressed fiber quality; however, further multi-year and multi-location studies are required to determine the stability and environmental consistency of these responses.

Future investigations should incorporate additional functional and technological parameters, including fiber digestibility, enzymatic accessibility, fermentation potential, and other quality characteristics relevant to biomass processing. Integrating these complementary approaches will enable a more comprehensive assessment of soybean pressed fiber and help identify variety–production-window combinations with potential suitability for specific green biorefinery applications.

Overall, our findings indicate that enzymatic hydrolysis of soybean pressed fiber into potentially fermentable oligosaccharide fractions represents a promising strategy for the sustainable valorization of soybean press cake within integrated green biorefinery systems and for the potential production of functional food ingredients. Further studies are warranted to evaluate the fermentability, bioavailability, and potential physiological effects of these oligosaccharide fractions.

## Figures and Tables

**Table 1:** Primary value measuring parameters of the stem of soybean varieties. Mean (±SD) values of stem anatomical traits measured in four soybean varieties (Bólyi 612, Pannónia, Isidor and Advisor). Different lowercase letters above the bars indicate significant differences among varieties according to Tukey’s honestly significant difference (HSD) post hoc test following one-way ANOVA (*p* < 0.05). (*n* = 3); ADV = Advisor; BOL = Bólyi 612; ISI = Isidor; PAN = Pannónia kincse.

Soybean Varieties	Stem Diameter (µm)	Pith Diameter (µm)	Thickness of Primary Cortex (µm)
ADV	4920.95 ± 380.4 ^a^	4515.1 ± 569.43 ^a^	124.44 ± 16.05 ^a^
BOL	4775.00 ± 312.3 ^a^	4449.03 ± 332.57 ^a^	110.19 ± 7.20 ^b^
ISI	4548.09 ± 420.9 ^a^	4102.73 ± 333.28 ^a^	117.31± 13.13 ^ab^
PAN	4652.22 ± 220.7 ^a^	4395.96 ± 661.82 ^a^	116.67 ±10.47 ^ab^

**Table 2:** The micro- and macro-element content of pressed fiber of soybean varieties during the first harvest. Data are presented as mean ± standard deviation (SD). Different letters indicate significant differences according to one-way ANOVA followed by Tukey’s HSD test (*p* < 0.05). (*n* = 3); ADV = Advisor; BOL = Bólyi 612; ISI = Isidor; PAN = Pannónia kincse.

mg/ kg	ADV			BOL			ISI			PAN		
B	27.21	±	3.34 ^a^	31.34	±	1.67 ^a^	27.77	±	3.42 ^a^	27.97	±	1.07 ^a^
Ba	13.12	±	1.50 ^a^	15.84	±	2.02 ^a^	13.19	±	0.54 ^a^	13.16	±	1.07 ^a^
Ca	9804.5	±	1111.91 ^b^	12,295.59	±	957.20 ^a^	10,152.18	±	371.95 ^ab^	10,281.92	±	945.79 ^ab^
Cu	6.64	±	0.21 ^ab^	7.20	±	0.44 ^a^	6.43	±	0.25 ^ab^	4.91	±	0.51 ^b^
Fe	491.97	±	229.95 ^a^	719.35	±	362.93 ^a^	622.49	±	213.89 ^a^	359.89	±	112.65 ^a^
K	7736.9	±	828.95 ^a^	6542.01	±	180.21 ^a^	6951.15	±	702.87 ^a^	6567.96	±	548.35 ^a^
Mg	1724.8	±	205.48 ^a^	2115.66	±	165.56 ^a^	1818.33	±	103.96 ^a^	1648.68	±	246.48 ^a^
Mn	88.77	±	21.15 ^ab^	116.95	±	25.00 ^a^	93.72	±	6.52 ^ab^	61.29	±	12.41 ^b^
Mo	1.70	±	0.15 ^a^	1.15	±	0.37 ^a^	1.34	±	0.36 ^a^	0.35	±	0.07 ^b^
Na	8.29	±	1.44 ^a^	9.51	±	2.21 ^a^	12.35	±	4.27 ^a^	9.96	±	2.21 ^a^
P	1846.5	±	178.07 ^a^	1930.42	±	88.45 ^a^	1598.94	±	226.97 ^a^	1780.29	±	155.73 ^a^
S	866.96	±	88.31 ^a^	923.47	±	50.14 ^a^	815.23	±	96.22 ^a^	821.88	±	87.06 ^a^
Sr	80.60	±	11.46 ^a^	99.59	±	7.75 ^a^	77.72	±	9.63 ^a^	84.68	±	7.30 ^a^
Zn	22.85	±	4.89 ^a^	22.92	±	1.15 ^a^	18.99	±	2.65 ^a^	22.57	±	2.16 ^a^

**Table 3:** The micro- and macro-element content of pressed fiber of soybean varieties during the second harvest. Data are presented as mean ± standard deviation (SD). Different letters indicate significant differences according to one-way ANOVA followed by Tukey’s HSD test (*p* < 0.05). (*n* = 3); ADV = Advisor; BOL = Bólyi 612; ISI = Isidor; PAN = Pannónia kincse.

mg/kg	ADV			BOL			ISI			PAN		
B	29.07	±	1.62 ^a^	31.07	±	0.94 ^a^	31.63	±	2.63 ^a^	30.26	±	0.48 ^a^
Ba	14.08	±	0.84 ^a^	14.31	±	0.36 ^a^	13.32	±	2.18 ^a^	14.54	±	0.69 ^a^
Ca	11,520.63	±	748.08 ^a^	12,161.08	±	500.45 ^a^	11,070.87	±	1232.84 ^a^	11,660.09	±	814.48 ^a^
Cu	5.93	±	0.25 ^a^	5.32	±	0.18 ^a^	5.41	±	0.37 ^a^	5.26	±	0.15 ^a^
Fe	316.14	±	91.95 ^a^	236.75	±	17.76 ^a^	213.55	±	68.53 ^a^	242.86	±	27.66 ^a^
K	8957.59	±	236.72 ^a^	6680.69	±	33.74 ^b^	7873.51	±	983.66 ^ab^	8371.67	±	243.80 ^a^
Mg	2217.03	±	54.88 ^a^	2153.96	±	142.96 ^a^	2208.35	±	87.74 ^a^	2248.13	±	127.49 ^a^
Mn	126.97	±	3.82 ^ab^	136.80	±	17.32 ^a^	135.71	±	5.32 ^a^	102.96	±	9.78 ^b^
Mo	0.82	±	0.09 ^a^	0.32	±	0.01 ^b^	0.66	±	0.28 ^ab^	0.38	±	0.09 ^b^
Na	22.56	±	1.53 ^a^	22.07	±	2.80 ^a^	21.37	±	6.44 ^a^	21.46	±	2.86 ^a^
P	1871.65	±	161.58 ^ab^	1688.97	±	57.59 ^a^	1727.06	±	125.11 ^a^	2069.99	±	113.54 ^b^
S	997.85	±	81.53 ^a^	933.90	±	36.08 ^a^	935.07	±	55.34 ^a^	995.96	±	15.73 ^a^
Sr	86.46	±	6.00 ^a^	94.19	±	3.89 ^a^	89.98	±	5.42 ^a^	87.57	±	4.89 ^a^
Zn	18.36	±	4.12 ^a^	14.24	±	1.46 ^a^	15.43	±	1.31 ^a^	19.56	±	2.64 ^a^

**Table 4:** Experimental field layout showing the arrangement of soybean varieties and plots.

	**1.13 m**	**0.4 m**	**1.13 m**	**0.4 m**	**1.13 m**	**0.4 m**	**1.13 m**	**0.4 m**	**1.13 m**	**0.4 m**	**1.13 m**	**0.4 m**	**1.13 m**	**0.4 m**	**1.13 m**	**0.4 m**	**1.13 m**	**0.4 m**	**1.13 m**	**0.4 m**	**1.13 m**	**0.4 m**	**1.13 m**	**0.4 m**	**1.13 m**
**4 m**	**3/3 Isidor**	path	**1/3 Advisor**	path	**4/3 Pannónia kincse**	path	**2/3 Bólyi 612**	path	**4/2 Pannónia kincse**	path	**3/2 Isidor**	path	**2/2 Bólyi 612**	path	**1/2 Advisor**	path	**2/1 Bólyi 612**	path	**4/1 Pannónia kincse**	path	**1/1 Advisor**	path	**3/1 Isidor**	path	**3/4 Isidor**

**Table 5:** Soil characteristics of the study site.

Parameter	Value
pH (KCL)	6.8
Plasticity index (y_1_)	5
Plasticity index (y_2_)	8
AL-extractable P_2_O_5_ (mg kg^−1^)	100
AL-extractable K_2_O (mg kg^−1^)	165
Humus content (%)	2.57
Arany plasticity index (KA)	42

## Data Availability

The raw data supporting the conclusions of this article will be made available by the authors on request.
